# Beyond ownership: the critical role of digital literacy in shaping the impact of digital access on physical activity

**DOI:** 10.3389/fpubh.2025.1718387

**Published:** 2026-01-12

**Authors:** Wangjie Li, Qi Ling

**Affiliations:** 1 Nanyang Normal University, Nanyang, China; 2 Guangxi Minzu University, Guangxi, China

**Keywords:** causal inference, China, digital access, digital literacy, moderated mediation, physical activity, suppression effect, urban–rural disparity

## Abstract

**Background:**

The rapid proliferation of digital technology is often assumed to automatically foster healthier lifestyles, yet its impact on physical activity (PA) remains inconsistent and poorly understood. Moving beyond the simplistic focus on access, this study proposes and tests a theoretically grounded moderated mediation framework. It posits that digital literacy is the pivotal mechanism through which access influences PA, and that this mediation process is fundamentally moderated by the urban–rural divide, a key structural inequalities in China.

**Methods:**

Utilizing nationally representative data from the 2022 China Family Panel Studies (*N* = 18,251), we tested this framework using a counterfactual-based causal mediation analysis, complemented by instrumental variable and robustness checks.

**Results:**

The analysis reveals a stark urban–rural heterogeneity in mechanisms. For urban residents, digital literacy served as a significant partial mediator. In stark contrast, a consistent suppression effect was identified among rural residents: digital access exerted a significant negative direct effect on PA, which was counterbalanced by a stronger positive indirect effect through digital literacy. This indicates that in rural contexts, mere access may inadvertently displace physical activity, and its net benefit is contingent entirely on digital literacy.

**Conclusion:**

Our findings challenge the techno-optimistic narrative that equates access with positive outcomes. They necessitate a strategic policy pivot—from infrastructure expansion to an integrated approach that concurrently provides access, builds contextualized digital capability, and shapes healthier digital environments, with interventions tailored to the distinct mediation and suppression mechanisms observed across settings.

## 1 Introduction

The global surge in digital technology penetration has profoundly reshaped various aspects of human life, including health behaviors and promotion (1, 2). Physical activity (PA), a cornerstone of public health, is no exception. A growing body of research has explored the potential of digital devices, particularly smartphones, and mobile applications (apps) to facilitate and increase participation in PA (3, 4). Numerous studies have established a positive correlation between digital access—ownership of smartphones, internet connectivity—and engagement in health-related activities, suggesting that technology can lower barriers to information and resources (5, 6).

However, the simplistic equation of “digital access = improved outcomes” is increasingly being challenged (7, 8). The mere ownership of a device does not guarantee its effective use for health promotion purposes. This gap between access and effective utilization points to the concept of digital literacy, broadly defined as the ability to use information and communication technologies to find, evaluate, create, and communicate information, requiring both cognitive and technical skills (9, 10). While the digital divide in terms of access is narrowing in many regions, a second-level digital divide, characterized by disparities in skills and literacy, is becoming more pronounced and critical (11, 12). This is particularly evident in the context of global health disparities, where socioeconomic status influences both access and capability (13).

Within the domain of physical activity, prior research has often focused on either access or literacy in isolation. Studies have shown that individuals with higher digital literacy are more likely to use technology for health information seeking and to adopt healthier behaviors (14, 15). Conversely, a lack of digital skills can exacerbate health inequalities, even among those with physical access to technology (16, 17). Yet, the interplay between digital access and digital literacy, and how they jointly influence PA, remains underexplored. Specifically, the mechanism through which access translates (or fails to translate) into action is not well understood. This raises a critical question: Does digital access alone drive increased physical activity, or is digital literacy the pivotal factor that unlocks the potential of such access?

The Chinese context provides a compelling natural experiment to investigate this question. China’s digital landscape is vast, with 1.092 billion internet users, and internet activity is overwhelmingly mobile-centric: 99.9% of users access the internet via smartphones (18). However, significant disparities in digital literacy persist, especially between urban and rural populations, driven by differences in education, income, and opportunities for skill development (19, 20). Urban residents often exhibit higher digital literacy, leveraging technology for a wider range of advanced activities, including health management (21). In contrast, rural residents, despite gaining improved digital access, may lack the necessary skills to utilize these tools effectively for purposes beyond basic communication and entertainment (19, 22, 23). This urban–rural dichotomy offers a unique opportunity to test the nuanced relationship between access, literacy, and behavioral outcomes.

**Figure 1:**
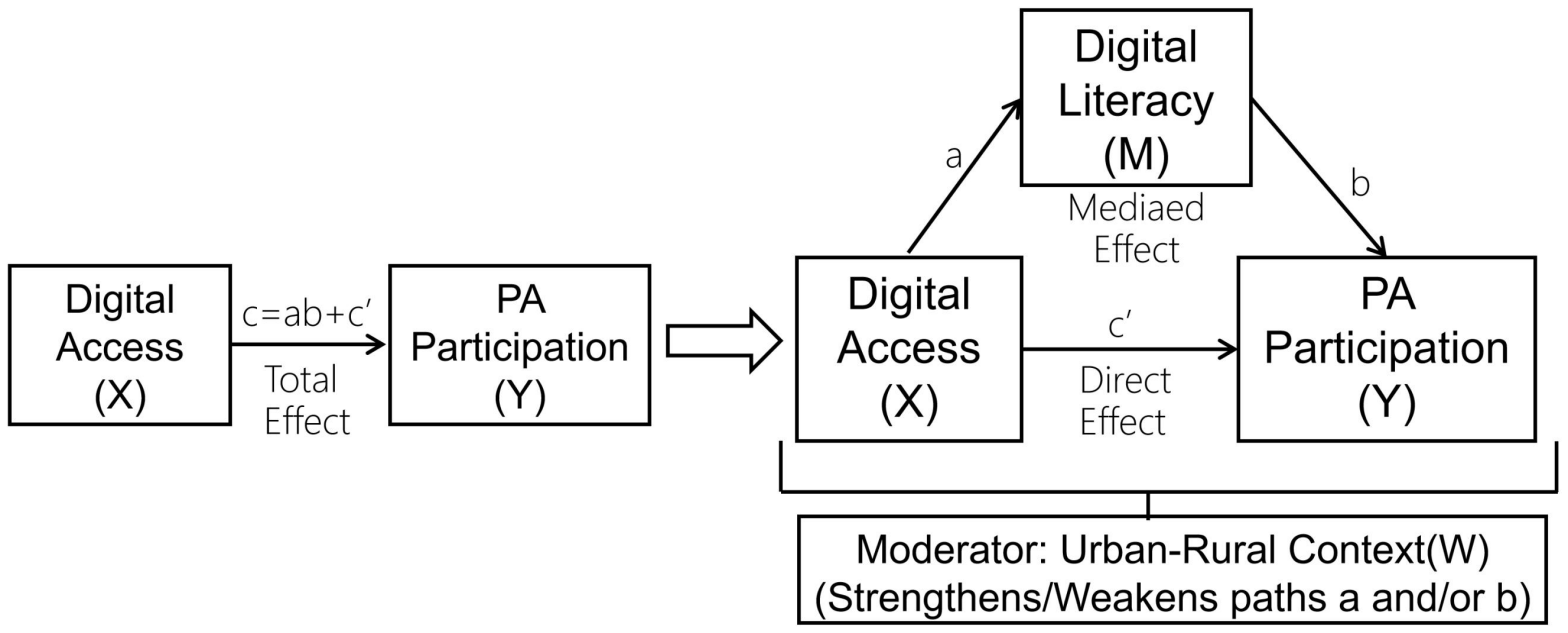
Hypothesized moderated mediation model. The conceptual model illustrating the proposed relationships. The effect of digital access (X) on physical activity (Y) is mediated by digital literacy (M). This mediation process is hypothesized to be moderated by the urban–rural context (W), meaning the strength of the a and/or b paths differs between urban and rural populations. Path c’ represents the direct effect of X on Y.

Guided by the second-level digital divide theory (11, 12) and social cognitive theory (24), this study proposes a moderated mediation framework to disentangle the mechanisms linking digital access to physical activity (see Figure 1). We posit that digital literacy serves as the pivotal mediating mechanism that translates digital access into behavioral change. Furthermore, we argue that the socioeconomic and infrastructural context, operationalized by the urban–rural divide, acts as a critical moderator that shapes the strength of this mediation pathway.

To empirically test this framework, we employ a nationally representative dataset from China, robust mediation analysis techniques, and an instrumental variable approach to address endogeneity. This study contributes to the literature by: (1) proposing and testing a theoretically-grounded moderated mediation model that explains how (via digital literacy) and when (under which urban–rural contexts) digital access influences PA; (2) providing empirical evidence on the nuanced mechanisms, including potential suppression effects, that underlie this relationship; and (3) deriving targeted policy implications that emphasize building digital capability alongside expanding digital infrastructure.

## 2 Materials and methods

### 2.1 Data source, study design, and ethical considerations

This study utilizes data from the 2022 wave of the China Family Panel Studies (CFPS), a nationally representative longitudinal survey administered by Peking University’s Institute of Social Science Survey (ISSS). The CFPS employs a multi-stage probability-proportional-to-size (PPS) sampling design with implicit stratification, covering 25 provincial-level administrative units across mainland China. It systematically collects comprehensive micro-level data on demographics, education, health, economic status, and digital technology use.

Our analytical sample was constructed by merging individual and household questionnaires. We retained respondents who provided information on both digital technology use and physical exercise. After listwise deletion of observations with missing values on core variables (digital access, digital literacy, physical activity, and hukou status), the final sample consisted of 18,251 individuals. The missing rates for all variables are presented in Appendix A. The PPS sampling design ensures the national representativeness of the data.

The sample was stratified into urban and rural residents based on the household registration (hukou) type to test the hypothesized moderated mediation (see Introduction, Figure 1). Respondents were classified as urban (hukou = 1) if their status was Non-Agricultural Hukou or Unified Resident Hukou, and as rural (hukou = 0) if their status was Agricultural Hukou. This stratification resulted in a final sample comprising 29.7% urban and 70.3% rural respondents.

The use of hukou status for stratification is based on its role as a fundamental institutional determinant of socioeconomic opportunity and lifestyle in China. While we acknowledge the phenomenon of hukou-residence mismatch, hukou remains a stable, legally defined marker of an individual’s long-term entitlement to public resources and is a well-established proxy for capturing structural urban–rural divides in Chinese social science research. Furthermore, the analysis does not employ sample weighting to balance the urban–rural ratio. This is because our primary analytical goal is to compare the underlying mechanisms (i.e., mediation patterns) between these structurally defined groups, rather than to estimate nationally representative population parameters. The regression models condition on key socioeconomic covariates (e.g., education, income) to account for compositional differences within this framework.

As a secondary analysis of de-identified public data from the CFPS-2022, this study was exempt from further ethical review under our institutional guidelines. The primary data collection for the CFPS was approved by the Institutional Review Board (IRB) at Peking University (Approval No.: IRB00001052-14010), with written informed consent obtained from all participants (25).

### 2.2 Variable specification

#### 2.2.1 Physical activity participation

A multidimensional variable system was constructed to assess physical activity participation, measured across three dimensions based on self-reported behavior over the past 12 months:

1. Frequency: ordinal scale (0 = never; 1 = <1/month; 2 = ≥1/month but <1/week; 3 = 1–2/week; 4 = 3–4/week; 5 = ≥5/week; 6 = daily; 7 = ≥2/day).
2. Duration: continuous measure (minutes per session).
3. General Participation: binary indicator (1 = engaged in at least one session in the past year; 0 = otherwise).

#### 2.2.2 Digital access and digital literacy

Digital access was proxied by two dummy variables indicating whether the respondent used a computer or a mobile device/tablet to access the internet. A composite indicator (value = 1 if either mode was used; 0 otherwise) captured overall access.

Digital literacy was measured as a composite index derived from multiple skill- and usage-related items across five dimensions, weighted using the entropy method following established approaches (26, 27). The specific measurement items, scoring rules, and their respective weights are detailed in Table 1 (comprehensive variable construction details are provided in the Appendix B). The resulting standardized index ranges from 0 to 1, with higher values indicating greater proficiency.

**Table 1:** Measurement framework of digital literacy indicators.

Dimension	Measurement items	Scoring	Weight
Digital usage	Internet use via mobile and computer devices	Combined duration of mobile and computer internet usage	0.097
Digital learning Digital social	Importance of internet for learning	Importance rated 1–5, very important = 5, very unimportant = 1	0.042
	Online learning behavior	Daily online learning = 2, online learning = 1, no = 0	0.200
	Importance of internet for family and friend contact	Importance rated 1–5, very important = 5, very unimportant = 1	0.038
	Use of WeChat	Yes = 1; No = 0	0.039
	Frequency of sharing on WeChat moments	Scale from 1 (never) to 7 (almost daily)	0.113
Digital work	Importance of internet for work	Importance rated 1–5, very important = 5, very unimportant = 1	0.045
Digital life	Importance of internet for daily life	Importance rated 1–5, very important = 5, very unimportant = 1	0.045
	Importance of internet for leisure and entertainment	Importance rated 1–5, very important = 5, very unimportant = 1	0.043
	Online shopping	Daily shopping = 2, occasional shopping = 1, no = 0	0.119
	Watching short videos	Daily watching = 2, occasional watching = 1, no = 0	0.015
	Online gaming	Daily gaming = 2, occasional gaming = 1, no = 0	0.204

It is important to note the conceptual and operational distinction between the core variables. Digital Access is a binary variable indicating the means to participate in the digital world. Digital Literacy is a continuous index measuring the competency and diversity of usage among those with access. While the literacy index includes a component for internet usage duration, this measures the intensity of use and is combined with 11 other items capturing skill application across different domains. This ensures that the mediator captures engaged capability, not merely the presence of access, allowing for the estimation of its distinct mediating role.

#### 2.2.3 Control variables

To mitigate omitted-variable bias, multilevel confounders were controlled. Individual-level variables included sociodemographics (hukou, age, gender), socioeconomic status (education, marital status, log household income), and health conditions (self-rated health, chronic disease status) (28). Subjective social status was incorporated given its documented association with health behaviors net of objective measures (29, 30), including evidence from China linking it to physical activity via psychosocial pathways (31). Regional fixed effects accounted for spatial heterogeneity (32).

All variable definitions and descriptive statistics are summarized in Table 2.

**Table 2:** Descriptive statistics for key variables.

Continuous variables	Meaning	Values	Mean	SD	Min	Max
Exercise frequency	Frequency of physical exercise participation	0 (never) to 7 (twice daily)	1.824	2.373	0	7
Exercise duration	“On average, how many minutes do you exercise each time (1–300)?”	Minutes per session	23.26	34.889	0	270
Exercise participation	Participation in physical exercise (dichotomous)	0 (never), 1 (any)	0.437	0.496	0	1
Digital access	Access to digital services	0 (no access), 1 (access)	0.737	0.440	0	1
Digital literacy	Entropy-weighted index (12 items)	Standardized index (0–1)	0.316	0.186	0.012	0.987
Hukou	Household registration type	0 (rural), 1 (urban)	0.297	0.457	0	1
Gender	Respondent’s gender	0 (female), 1 (male)	0.503	0.5	0	1
Age	Respondent’s age in years	Years	45.218	16.741	16	97
Partnered	Currently living with a romantic partner (married or cohabiting)	0 (solo), 1 (paired)	0.751	0.432	0	1
Years of education	Completed years of formal education	Years of formal education	9.44	4.688	0	23
Ln household income	Natural log of per capita household income (yuan) (CNY)	Log of per capita household income	10.004	1.078	0	15.745
Subjective social status	On a scale of 1–5, where do you place your social status locally?	1 (very low) to 5 (very high)	3.01	1.052	1	5
Self-rated health	Overall health	1 (worst) to 5 (best)	3.141	1.168	1	5
Chronic disease	Chronic disease diagnosis (past 6 months)	0 (no), 1 (yes)	0.155	0.362	0	1

Categorical variable	Meaning	Category	*N*	%
Region	Geographic region	East	7,665	42.00%
		Central	5,509	30.18%
		West	5,077	27.82%

Continuous variables are reported as Mean ± SD, together with *N*, Min, and Max. Binary variables are reported as Mean ± SD where the mean equals the proportion. All statistics are based on the analytic sample (*N* = 18,251).

### 2.3 Model specification and causal mediation analysis

To rigorously examine the mediating role of digital literacy (M) in the relationship between digital access (X) and physical activity (Y), and to test the hypothesized moderated mediation by the urban–rural context, we employed a causal mediation analysis based on the counterfactual framework (33, 34). This method allows for the formal decomposition of the total effect (TE) of digital access into two components:

The Average Causal Mediation Effect (ACME), which represents the average indirect effect operating through the mediator, digital literacy. The Average Direct Effect (ADE), which represents the average effect of digital access on physical activity not transmitted through the proposed mediator. We estimated the ACME, ADE, and TE along with their 95% confidence intervals using a quasi-Bayesian Monte Carlo method based on 1,000 simulations. The presence of a significant mediation effect is established if the 95% confidence interval for the ACME does not contain zero. A suppression effect (or inconsistent mediation) is identified when the ACME and ADE exhibit statistically significant effects in opposite directions.

A sensitivity analysis is recommended within this framework to assess the robustness of the ACME to potential violations of the sequential ignorability assumption, which requires no unmeasured confounding of the mediator-outcome relationship (34). However, due to computational constraints and high memory demands when applying the sensitivity analysis to our large full sample (*N* = 18,251), convergence could not be achieved for the full dataset.

To address this and provide evidence for the robustness of our findings, we conducted a supplementary analysis on a randomly selected 50% subsample of the full data. The results of the causal mediation analysis and the successful sensitivity analysis from this subsample are presented in Appendix Table C1. The core findings regarding the significance and directions of the ACME and ADE are consistent with those from the full sample analysis reported in the main text. This consistency, coupled with the sensitivity results from the subsample, provides strong confidence in the robustness of our conclusions.

All analyses were performed using Stata 17, with the medeff and medsens commands employed for the causal mediation analysis and sensitivity analysis, respectively.

## 3 Results

### 3.1 Descriptive statistics

Table 2 reports the variable definitions and basic descriptive statistics. A preliminary analysis of group differences, as detailed in Table 3, reveals that all major variables differ significantly (*p* < 0.01) between urban and rural residents. These findings underscore the pronounced socioeconomic and behavioral disparities in contemporary China, establishing the necessary context for the mediation analysis that follows.

**Table 3:** Urban–rural differences in key variables.

Variable	Rural (*N* = 12,823)	Urban (*N* = 5,428)	MeanDiff	*P* value	Effect size	Effect size type
Continuous variables	(Mean ± SD)	(Mean ± SD)	(Urban–Rural)		[95% CI]	
Exercise frequency	1.491 ± 2.237	2.610 ± 2.497	1.118	<0.01	−0.483 [−0.515, −0.451]	Cohen’s *d*
Exercise duration	18.728 ± 32.556	33.964 ± 37.762	15.236	<0.01	−0.446 [−0.478, −0.414]	Cohen’s *d*
Digital literacy	0.293 ± 0.182	0.369 ± 0.185	0.076	<0.01	−0.415 [−0.447, −0.383]	Cohen’s *d*
Age	44.863 ± 16.670	46.056 ± 16.880	1.193	<0.01	−0.071 [−0.103, −0.040]	Cohen’s *d*
Years of education	8.457 ± 4.618	11.761 ± 3.981	3.303	<0.01	−0.744 [−0.777, −0.712]	Cohen’s *d*
Ln household income	9.793 ± 1.045	10.502 ± 0.987	0.709	<0.01	−0.690 [−0.722, −0.657]	Cohen’s *d*
Subjective social status	3.050 ± 1.091	2.916 ± 0.947	−0.134	<0.01	0.127 [0.095, 0.159]	Cohen’s *d*
Self-rated health	3.162 ± 1.211	3.092 ± 1.058	−0.07	<0.01	0.060 [0.028, 0.092]	Cohen’s *d*

Binary variables	Rural (*N*, %)	Urban (*N*, %)		*p*-value	Effect size [95% CI]	Effect size type
Exercise participation	4,674 (36.5%)	3,299 (60.8%)		<0.01	0.243 [0.228, 0.259]	RD
Digital access	8,922 (69.6%)	4,527 (83.4%)		<0.01	0.138 [0.126, 0.151]	RD
Gender (male)	6,463 (50.4%)	2,709 (49.9%)		0.542	0.005 [−0.021, 0.011]	RD
Partnered	9,636 (75.2%)	4,075(75.1%)		0.918	0.0007 [−0.015, 0.013]	RD
Chronic disease	1,892 (14.8%)	946 (17.4%)		<0.01	0.027 [0.015, 0.039]	RD

Categorical variables	(*N*, %)	(*N*, %)	Chi-square	*p*-value	Effect size [95% CI]	Effect size type
Region			318.221	<0.01	*V* = 0.132	Cramer’s *V*
East	5,074 (39.57%)	2,591 (47.73%)				
Central	3,689 (28.77%)	1,820 (33.53%)				
West	4,060 (31.66%)	1,017 (18.74%)				

Effect size interpretation: Cohen’s *d*: negligible (<0.2), small (0.2–0.49), medium (0.5–0.79), large (≥0.8); Risk Difference (RD): Absolute difference in proportions (Urban–Rural); Cramer’s *V*: weak (0.1–0.29), moderate (0.3–0.49), strong (≥0.5). Sample size consideration: The rural-to-urban ratio (2.36:1) increases power to detect smaller effects in rural comparisons, but effect size metrics provide sample-size-independent measures of practical significance.

Urban residents reported substantially higher levels of physical activity participation compared to their rural counterparts. The average exercise frequency was 2.61 (SD = 2.50) for urban samples, equivalent to approximately 1–2 sessions per week, which is 75% higher than the rural average of 1.49 (SD = 2.24). A similar gap was evident in exercise duration, with urban residents exercising for an average of 33.96 (SD = 37.89) minutes per session, nearly double the 18.73 (SD = 32.65) minutes reported by rural residents. The disparity was also stark in the binary measure of participation, with 60.8% of urban residents engaging in exercise versus 36.5% of rural residents.

Consistent with these behavioral patterns, significant divides were found in digital indicators. Digital access was 13.8 percentage points higher in urban areas (83.4% vs. 69.6%). More critically, the composite digital literacy index averaged 0.369 (SD = 0.19) for urban residents, significantly higher (*p* < 0.01) than the rural average of 0.293 (SD = 0.18), confirming a substantial gap in digital capability alongside access (35). These digital disparities are mirrored by significant socioeconomic differences, such as a 3.3-year gap in educational attainment and a higher logarithm of household income in urban areas.

These baseline disparities highlight the deeply stratified contexts of digital exposure and health behaviors in China, setting the stage for a nuanced analysis of how digital access and literacy interact to shape physical activity outcomes within these divergent environments.

### 3.2 Regression and causal mediation results for the full sample

To elucidate the mechanism linking digital access to physical activity, we present both stepwise regression coefficients and formal causal mediation analysis in Table 4. The causal mediation analysis, based on the Imai et al. Framework (33, 34), provides robust estimates of the Average Causal Mediation Effect (ACME, indirect effect) and the Average Direct Effect (ADE) for the full sample (*N* = 18,251).

**Table 4:** Causal mediation analysis of the effect of digital access on physical activity via digital literacy.

Model variable	(1) Digital literacy	(2) Exercise frequency	(3) Exercise duration
Stepwise regression coefficients			
Digital access	0.189^***^ (0.002)	0.036 (0.056)	−1.900^**^ (0.811)
Digital literacy	–	1.582^***^ (0.147)	27.190^***^ (2.144)
Hukou	0.020^***^ (0.002)	0.682^***^ (0.041)	9.331^***^ (0.595)
Causal mediation analysis (Imai et al. (33, 34) Framework)			
ACME (Indirect effect)	–	0.301 [0.252, 0.355]	5.144 [4.335, 5.935]
ADE (Direct effect)	–	0.032 [−0.074, 0.135]	−1.885 [−3.418, −0.330]
Total effect	–	0.333 [0.240, 0.425]	3.259 [1.901, 4.643]
Proportion mediated	–	90.5% [0.708, 1.253]	157.9% [1.108, 2.706]
Model information			
Controls	Yes	Yes	Yes
Regional effects	Yes	Yes	Yes
_cons	0.133^***^ (0.010)	−1.823^***^ (0.197)	−18.468^***^ (2.808)
*N*	18,251	18,251	18,251
*r* ^2^	0.6598	0.1010	0.1137

ACME, Average Causal Mediation Effect (indirect effect); ADE, Average Direct Effect.

^***^p < 0.01; ^**^p < 0.05; ^*^p < 0.1. Robust standard errors in parentheses for regression coefficients. 95% confidence intervals in brackets for causal mediation analysis, based on the Imai et al. (34) framework with 1,000 simulations. All models include full controls for sociodemographics, socioeconomic status, health conditions, and regional fixed effects.

#### 3.2.1 Exercise frequency

The causal mediation analysis provides robust evidence for a strong mediating effect. The Average Causal Mediation Effect (ACME), representing the indirect effect through digital literacy, is positive and statistically significant (ACME = 0.301, 95% CI [0.252, 0.355]). In contrast, the Average Direct Effect (ADE) of digital access is negligible and not statistically significant (ADE = 0.032, 95% CI [−0.074, 0.135]). The Total Effect of digital access is positive and significant (0.333, 95% CI [0.240, 0.425]). Digital literacy mediates approximately 90.5% of the total effect of digital access on exercise frequency. This pattern, characterized by a significant ACME and a non-significant ADE, indicates that digital access influences exercise frequency almost entirely through its effect on digital literacy, supporting a case of full mediation.

#### 3.2.2 Exercise duration

For exercise duration, the analysis reveals a more complex suppression effect.^1^ The ACME is positive and significant (5.144, 95% CI [4.335, 5.935]), while the ADE is significant and negative (−1.885, 95% CI [−3.418, −0.330]). The opposing signs of the ADE and ACME confirm a classic suppression pattern. The positive Total Effect (3.259, 95% CI [1.901, 4.643]) emerges because the strong, positive indirect effect via digital literacy masks or suppresses the underlying negative direct effect of digital access. The proportion mediated exceeds 100% (157.9%), which is a mathematical consequence of suppression (when direct and indirect effects have opposing signs). This finding indicates that without the empowering role of digital literacy, digital access would have a net negative impact on exercise duration.

Robustness to Unobserved Confounding. To assess the sensitivity of these mediation findings to potential unobserved confounders, we conducted a supplementary analysis on a randomly selected 50% subsample (*N* = 9,125), which enabled the computationally intensive sensitivity analysis. As detailed in Appendix Table C1, the results for this subsample are highly consistent with the full-sample estimates for both ACME and ADE. Crucially, the sensitivity analysis indicates that an unobserved confounder would need to be substantially correlated with both the mediator and outcome (*ρ* > 0.078 for frequency; *ρ* > 0.093 for duration) to nullify the observed mediation effects. These relatively high thresholds bolster confidence that our core findings are not easily explained by omitted variable bias.

Overall, these findings underscore the pivotal role of digital literacy. The causal mediation analysis confirms that it is not mere access to digital technology, but rather the ability to use it effectively, that drives increases in physical activity. For exercise frequency, it is the primary channel; for exercise duration, it is a critical counterweight that reverses the otherwise negative direct impact of access.

### 3.3 Urban–rural heterogeneity: causal mediation analysis by sub-samples

Table 5 presents the results of the causal mediation analysis stratified by urban–rural residence, revealing profound contextual heterogeneity in the mechanisms linking digital access to physical activity. The integrated sensitivity analysis further clarifies the robustness of these findings to potential unobserved confounding.

**Table 5:** Urban–rural differences in causal mediation effect of digital access on physical activity via digital literacy.

Model variable	Urban			Rural		
	(1) Digital literacy	(2) Exercise frequency	(3) Exercise duration	(4) Digital literacy	(5) Exercise frequency	(6) Exercise duration
Stepwise regression coefficients						
Digital access	0.211^***^ (0.005)	0.311^***^ (0.118)	2.436^**^ (1.794)	0.183^***^ (0.003)	−0.164^***^ (0.063)	−4.138^***^ (0.897)
Digital literacy	–	0.616^**^ (0.147)	15.927^***^ (4.035)	–	2.155^***^ (0.176)	33.342^***^ (2.523)
Causal mediation analysis (Imai et al. (33, 34) Framework)						
ACME (Indirect effect)	–	0.130 [0.020, 0.239]	3.366 [1.704, 5.017]	–	0.394 [0.329, 0.457]	6.089 [5.163, 6.999]
ADE (Direct effect)	–	0.313 [0.090, 0.539]	2.471 [−0.922, 5.909]	–	−0.163 [−0.281, −0.043]	−4.121 [−5.818, −2.402]
Total effect	–	0.443 [0.243, 0.652]	5.837 [2.789, 9.007]	–	0.231 [0.126, 0.337]	1.968 [0.472, 3.504]
Proportion mediated	–	29.4% [0.200, 0.536]	57.7% [0.374, 1.207]	–	170.5% [1.167, 3.114]	308.5% [1.721, 11.944]
Sensitivity results (95% Confidence interval)						
Rho at which ACME = 0		0.0313	0.0534	–	0.1068	0.1154
R^2^_M * R^2^_Y* at which ACME = 0		0.001	0.0029	–	0.0114	0.0133
R^2^_M ~ R^2^_Y~ at which ACME = 0		0.0004	0.0011	–	0.0035	0.0039
Model information						
Controls	Yes	Yes	Yes	Yes	Yes	Yes
Regional effects	Yes	Yes	Yes	Yes	Yes	Yes
_cons	0.122^***^ (0.021)	−3.392^***^ (0.401)	−40.522^***^ (6.108)	0.147^***^ (0.012)	−0.643^***^ (0.231)	−6.828^***^ (3.311)
*N*	5,428	5,428	5,428	12,823	12,823	12,823
*r* ^2^	0.5856	0.0835	0.0709	0.6782	0.0601	0.0902

ACME, Average Causal Mediation Effect (Indirect Effect); ADE, Average Direct Effect.

*p* < 0.1, ^**^p < 0.05, ^***^p < 0.01. Robust standard errors in parentheses for stepwise regression. 95% confidence intervals in brackets for causal mediation analysis, based on the framework by Imai et al. (34) with 1,000 simulations. All models include full controls for sociodemographics, socioeconomic status, health conditions, and regional fixed effects.

#### 3.3.1 Urban sub-sample

Among urban residents, digital access significantly enhanced digital literacy (*β* = 0.211, *p* < 0.01). For exercise frequency, the analysis confirmed a partial mediation mechanism. A significant positive indirect effect was observed (ACME = 0.130, 95% CI [0.020, 0.239]), and the direct effect of digital access also remained positive and significant (ADE = 0.313, 95% CI [0.090, 0.539]). This indicates that digital access benefits urban residents’ exercise frequency both directly and by improving their digital literacy.

For exercise duration, the pattern differed. While the indirect effect through digital literacy was significant and substantial (ACME = 3.366, 95% CI [1.704, 5.017]), the direct effect was not statistically significant (ADE = 2.471, 95% CI [−0.922, 5.909]). This suggests that digital literacy plays a fully mediating roleexercise duration among urban residents.

The sensitivity analysis for the urban sample (Table 5) indicates that the mediation effects are robust. The estimated ACME for exercise frequency (*ρ* = 0.031) and duration (*ρ* = 0.053) would only be explained away by an unobserved confounder of non-trivial strength.

#### 3.3.2 Rural sub-sample

The mechanisms in rural areas were distinctly different and characterized by suppression effects for both outcomes. Digital access significantly increased digital literacy (*β* = 0.183, *p* < 0.01).

For exercise frequency, a significant negative direct effect was identified (ADE = −0.163, 95% CI [−0.281, −0.043]), which was counterbalanced by a significant positive indirect effect via digital literacy (ACME = 0.394, 95% CI [0.329, 0.457]). This results in a net positive total effect (0.231). The proportion mediated was 170.5%, confirming a strong suppression effect.

An even more pronounced suppression effect was found for exercise duration. The negative direct effect was large and significant (ADE = −4.121, 95% CI [−5.818, −2.402]), while the positive indirect effect was even larger (ACME = 6.089, 95% CI [5.163, 6.999]), leading to a smaller but positive total effect (1.968).

Critically, the sensitivity analysis for the rural sample (Table 5) reveals that the key suppression effects are highly robust to potential confounding. The estimated ACMEs for frequency (*ρ* = 0.107) and duration (*ρ* = 0.115) are particularly sturdy; an unmeasured confounder would need to be very strongly correlated with both the mediator and outcome to nullify these effects. This provides strong confidence in the conclusion that digital literacy is essential to unlock the positive potential of digital access in rural contexts.

In summary, the urban–rural heterogeneity is stark and robust. In urban areas, digital access exhibits a neutral-to-beneficial direct influence, with digital literacy acting as a partial or full mediator. In contrast, in rural areas, digital access itself carries a significant direct cost to physical activity, and its net benefit is contingent entirely upon digital literacy, which acts as a suppressor.

### 3.4 Endogeneity correction: IV-2SLS estimates of the mediation model

To address potential endogeneity concerns (e.g., reverse causality or omitted variable bias), we employed an Instrumental Variable Two-Stage Least Squares (IV-2SLS) approach. We used lagged digital access from the 2020 wave (“Digital Access 2020”) as an instrument for current digital access. This instrument satisfies the relevance condition, as historical access is strongly correlated with current usage (First-stage *F*-statistics are 383.59 for urban and 740.92 for rural, vastly exceeding the 16.38 benchmark, Table 6). It also plausibly satisfies the exclusion restriction, as past access is unlikely to affect current physical activity except through its influence on forming current digital habits, conditional on covariates.

**Table 6:** Instrumental variable estimates of the mediation model: The effect of digital access on physical activity via digital literacy.

Model variable	Urban				Rural			
	(1) Digital access	(2) Digital literacy	(3) Exercise frequency	(4) Exercise duration	(5) Digital access	(6) Digital literacy	(7) Exercise frequency	(8) Exercise duration
Digital access 2020	0.393^***^ (0.020)				0.344^***^ (0.013)			
Digital access		0.292^***^ (0.015)	0.551 (0.474)	−1.184 (6.659)		0.269^***^ (0.010)	0.060 (0.243)	−9.490^***^ (3.231)
Digital literacy			0.393 (0.470)	18.916^***^ (7.003)			2.070^***^ (0.324)	42.442^***^ (4.706)
First-stage *F* statistic	383.59^***^				740.92^***^			
Kleibergen–Paap LM test	48.06^***^	48.06^***^	41.76^***^	41.76^***^	100.64^***^	100.64^***^	65.74^***^	65.74^***^
Cragg–Donald Wald F	876.41	876.41	564.45	564.45	1468.22	1468.22	815.75	815.75
KP Wald F	383.59	383.59	243.11	243.11	740.92	740.92	358.66	358.66
Stock–Yogo 10% threshold	16.38	16.38	16.38	16.38	16.38	16.38	16.38	16.38
Controls	Yes	Yes	Yes	Yes	Yes	Yes	Yes	Yes
Regional effects	Yes	Yes	Yes	Yes	Yes	Yes	Yes	Yes
*N*	4,372	4,372	4,372	4,372	10,373	10,373	10,373	10,373

Models (1) and (5) are the first-stage regressions. Models (2)–(4) and (6)–(8) are the second-stage regressions of the IV-2SLS mediation model.

*p* < 0.1, ^**^p < 0.05, ^***^p < 0.01. Robust standard errors in parentheses. All models include full controls for sociodemographics, socioeconomic status, health conditions, and regional fixed effects. The Stock–Yogo weak ID test critical value for 10% maximal IV size is 16.38.

The exclusion restriction requires that past digital access affects current physical activity only through its influence on forming current digital habits and literacy, conditional on covariates. While major events like the COVID-19 pandemic between survey waves may have influenced overall activity levels, we argue that the primary channel through which 2020 access shapes 2022 behavior remains the digitally mediated pathway of skill and habit formation. The pandemic is unlikely to have systematically altered this specific historical pathway in a way that directly correlates with 2022 physical activity, after controlling for the observed covariates including health status and regional effects.

The IV estimates, which provide a causal lens to scrutinize the mechanisms, robustly confirm and sharpen the findings from the causal mediation analysis (Section 3.2 & 3.3).

For urban residents, the IV results reinforce the notion that digital literacy is the primary channel. The instrumented digital access has a strong, significant positive effect on digital literacy (*β* = 0.292, *p* < 0.01, Column 2). For exercise duration, its direct effect is insignificant (*β* = −1.184, Column 4), while digital literacy exhibits a large and significant positive effect (*β* = 18.916, *p* < 0.01). This pattern is consistent with the full mediation effect identified in Table 5, strengthening the conclusion that for urban residents, the benefit of digital access on exercise duration is fully realized by enhancing their digital capability.

For rural residents, the IV estimates unveil a more stark picture of the suppression effect. The direct effect of instrumented digital access on exercise duration is significantly negative and substantially larger in magnitude (*β* = −9.490, *p* < 0.01, Column 8) than the OLS and causal mediation estimates. Concurrently, the positive effect of digital literacy remains powerfully significant (*β* = 42.442, p < 0.01). This indicates that after purging potential endogeneity, the inherent time-displacing nature of digital technology in rural contexts is even more pronounced. However, the potent positive indirect pathway via digital literacy is also confirmed to be robust. A similar pattern of a negative (though insignificant) direct effect and a strong positive effect of literacy is observed for exercise frequency (Columns 6–7), aligning with the suppression effect found in the main analysis.

The IV mediation analysis provides robust causal evidence that the central role of digital literacy is not an artifact of endogeneity. The urban–rural heterogeneity is causal in nature: in urban areas, digital access works primarily by building literacy; in rural areas, it carries a significant direct cost to physical activity that can only be overcome, and transformed into a net benefit, through the powerful mediating channel of digital literacy.

### 3.5 Robustness checks

To ensure the robustness of our core findings, we subjected the moderated mediation model to a series of stringent tests using alternative model specifications and samples. The results of the causal mediation analysis across these tests are consolidated in Table 7. The findings consistently reaffirm the pivotal mediating role of digital literacy and the stark urban–rural heterogeneity, with the sensitivity analysis further confirming their robustness to potential unobserved confounding.

**Table 7:** Robustness checks: Causal mediation analysis under alternative specifications.

Model variable	(1)	(2)	(3)	(4)	(5)	(6)	(7)	(8)	(9)	(10)
	Urban	Rural	Urban	Working-age	Rural	Working-age	Non-municipality	Urban	Non-municipality	Rural
	Exercise participation	Exercise participation	Exercise frequency	Exercise duration	Exercise frequency	Exercise duration	Exercise frequency	Exercise duration	Exercise frequency	Exercise duration
Stepwise regression coefficients										
Digital access	0.126 (0.104)	−0.171^***^ (0.065)	0.281^**^ (0.138)	2.072 (2.181)	−0.144^**^ (0.064)	−3.991^***^ (0.936)	0.316^**^ (0.126)	3.554 (1.940)	−0.164^**^ (0.064)	−4.223^***^ (0.911)
Digital literacy	1.183^***^ (0.242)	2.492^***^ (0.177)	0.807^***^ (0.279)	18.023^***^ (4.417)	2.417^***^ (0.179)	33.223^***^ (2.617)	0.808^***^ (0.286)	16.142^***^ (4.396)	2.146^***^ (0.180)	33.496^***^ (2.570)
Causal mediation analysis (Imai et al. (33, 34) Framework)										
Average ACME (Indirect effect)	0.056 [0.033, 0.077]	0.093 [0.080, 0.106]	0.155 [0.052, 0.258]	3.466 [1.831, 5.099]	0.412 [0.350, 0.473]	5.667 [4.765, 6.556]	0.169 [0.052, 0.284]	3.365 [1.580, 5.139]	0.391 [0.326, 0.455]	6.102 [5.161, 7.027]
Average ADE (Direct effect)	0.029 [−0.015, 0.073]	−0.035 [−0.059, −0.009]	0.284 [0.024, 0.548]	2.114 [−2.011, 6.294]	−0.143 [−0.264, −0.020]	−3.973 [−5.744,-2.178]	0.318 [0.079, 0.561]	3.592 [−0.079, 7.311]	−0.162 [−0.283, −0.040]	−4.205 [−5.928, −2.459]
Total effect	0.084 [0.044, 0.125]	0.058 [0.036, 0.081]	0.439 [0.193, 0.696]	5.580 [1.651, 9.678]	0.269 [0.160, 0.383]	1.695 [0.096, 3.350]	0.487 [0.272, 0.712]	6.957 [3.650, 10.403]	0.229 [0.123, 0.337]	1.897 [0.378, 3.457]
Proportion mediated	66.1%[0.445, 1.252]	159.1%[1.148, 2.576]	35.6%[0.223, 0.803]	62.4%[0.355, 1.999]	153.2% [1.077, 2.578]	327.9% [1.487, 20.326]	34.6% [0.237, 0.620]	48.4%[0.323, 0.922]	170.9% [1.160, 3.189]	320.4%[1.727, 14.160]
Sensitivity results (95% Confidence interval)										
Rho at which ACME = 0	0.100	0.200	0.0436	0.0616	0.1295	0.1221	0.0414	0.054	0.1062	0.116
R^2^_M * R^2^_Y* at which ACME = 0	0.010	0.040	0.0019	0.0038	0.0168	0.0149	0.0017	0.0029	0.0113	0.0135
R^2^_M ~ R^2^_Y ~ at which ACME = 0	0.0026	0.0078	0.0009	0.0018	0.0064	0.0055	0.0007	0.0011	0.0034	0.004
Model information										
Controls	Yes	Yes	Yes	Yes	Yes	Yes	Yes	Yes	Yes	Yes
Regional effects	Yes	Yes	Yes	Yes	Yes	Yes	Yes	Yes	Yes	Yes
_cons	−4.548^***^ (0.382)	−3.025^***^ (0.246)	−3.612^***^ (0.441)	−44.216^***^ (6.988)	−1.000^***^ (0.247)	−10.719^***^ (3.616)	−4.075^***^ (0.451)	−50.487^***^ (6.917)	−0.571^***^ (0.236)	−5.734 (3.376)
*N*	5,428	12,823	4,354	4,354	10,566	10,566	4,586	4,586	12,340	12,340
*r* ^2^	0.0716	0.0930	0.0940	0.0858	0.0749	0.1080	0.0937	0.0744	0.0603	0.0910

ACME, Average Causal Mediation Effect (Indirect Effect); ADE, Average Direct Effect. *p* < 0.1, ^**^p < 0.05, ^***^p < 0.01. Robust standard errors in parentheses for stepwise regression. 95% confidence intervals in brackets for causal mediation analysis, based on the framework by Imai et al. (34) with 1,000 simulations. All models include full controls.

#### 3.5.1 Alternative measure of physical activity

We first replaced the continuous measures of physical activity with a binary indicator (exercise participation). The causal mediation results remained highly consistent with the main analysis. For urban residents, the indirect effect of digital access via digital literacy was positive and significant (ACME = 0.056, 95% CI [0.033, 0.077]), while the direct effect was insignificant (ADE = 0.029). For rural residents, we again identified a clear suppression effect: a significant positive indirect effect (ACME = 0.093) was coupled with a significant negative direct effect (ADE = −0.035). The sensitivity analysis confirms these effects are robust, particularly for the rural sample where the estimated ACME would require a substantial confounder (*ρ* = 0.200) to be nullified.

#### 3.5.2 Working-age sub-sample (18–64 years)

Restricting the analysis to the working-age population, our key findings held without exception. In urban areas, digital literacy served as a significant mediator for both frequency (ACME = 0.155) and duration (ACME = 3.466). The direct effect was positive and significant for frequency, indicating partial mediation, but insignificant for duration. In rural areas, the signature suppression effect was powerfully present for both outcomes, with significant negative ADEs for frequency (−0.143) and duration (−3.973) alongside large and significant ACMEs. The sensitivity parameters (*ρ* = 0.044–0.130 across samples) demonstrate that these mediation patterns are not easily explained by unobserved confounding.

#### 3.5.3 Regional heterogeneity: excluding municipalities

To mitigate potential bias from unique socioeconomic policies in major municipalities (Beijing, Shanghai, Tianjin, Chongqing), we excluded these regions. The results for the remaining provinces aligned closely with the full-sample analysis. For urban residents, the pattern of partial mediation for exercise frequency and full mediation for duration was replicated. For rural residents, the suppression effect remained robust for both outcomes. Notably, the sensitivity analysis shows consistently high *ρ* values (0.041–0.116) across these geographical subsamples, indicating that the urban–rural heterogeneity in mediation mechanisms is a robust finding.

Across all robustness checks—varying the outcome measure, sample composition, and geographic coverage—the core conclusions are unchallenged and in fact, are reinforced. The consistent and significant positive ACME underscores that digital literacy is a robust and fundamental mechanism linking digital access to physical activity. The comprehensive sensitivity analysis provides strong evidence that these findings are not easily attributable to unobserved confounding. Furthermore, the persistent finding of significant negative direct effects in rural contexts across nearly all specifications solidifies the evidence for a pervasive suppression effect.

#### 3.5.4 Robustness to model specification: ordered logit analysis for exercise frequency

To further assess the robustness of our findings, we examined whether the results for exercise frequency were sensitive to the choice of model specification. Given the ordered categorical nature of the exercise frequency variable, we re-estimated the models using ordered logit regression for the full, urban, and rural samples (see Appendix Tables D1–D3).

The results from these non-linear models fully corroborate our main conclusions. First, the coefficient for digital literacy remained positive and highly statistically significant (*p* < 0.01) across all samples, reaffirming its robust positive association with exercise frequency. Second, the pattern of urban–rural heterogeneity persisted. Notably, in the rural sub-sample, the direct coefficient of digital access became statistically insignificant in the ordered logit specification, which aligns with the full mediation and suppression patterns identified in our primary causal mediation analysis.

## 4 Discussion

This study provides robust evidence that digital literacy, rather than mere digital access, serves as the critical mechanism shaping physical activity outcomes in the digital era. By testing a moderated mediation model with causal inference methods, our findings reveal a nuanced and context-dependent picture. The mediating role of digital literacy is not only significant but its nature is fundamentally conditioned by the urban–rural context. Specifically, our causal mediation and IV analyses characterize this relationship as a mix of partial and full mediation in urban areas, contrasted with a pervasive suppression effect in rural areas. These results fundamentally challenge the techno-optimistic assumption that digital access alone can uniformly promote health behaviors (8), demonstrating that its net benefit is contingent upon individuals’ capability to effectively utilize digital resources, and in underserved contexts, access without literacy may even carry direct behavioral costs.

### 4.1 Key findings and theoretical implications

First, we advance beyond the first-level digital divide to illuminate how the second-level divide (literacy) is the key mechanism shaping health behavior inequalities (12). The robust suppression effect observed for rural residents critically extends van Dijk’s digital divide theory (7, 11) by revealing a context-dependent dual nature of digital access. We empirically demonstrate that in underserved settings, access without literacy does not merely yield null effects but can actively produce unintended negative behavioral consequences (likely via displacement), while concurrently holding a positive potential that remains locked. This indicates that the consequence of the digital divide is not a simple linear gradient from ‘have-nots’ to ‘haves,’ but can involve competing pathways where the net benefit of technology is contingent on localized capabilities. This nuance offers a compelling explanation for mixed findings in digital health research (36) and underscores the importance of contextual analysis. Second, our identification of pronounced urban–rural heterogeneity enriches the literature on health disparities. The complex mediation pattern in urban areas (partial for frequency, full for duration) aligns with social cognitive theory (24), suggesting digital literacy enhances self-efficacy while direct access may retain a role that varies by the type of activity. Conversely, the consistent rural suppression pattern reflects a context where rapid technology adoption may have outpaced the development of supportive skills and a health-promoting digital environment, creating clear behavioral trade-offs (19, 22). This contextualization responds to calls for more spatially nuanced analyses of digital health impacts (37). by employing a unified causal inference framework—combining causal mediation analysis with an instrumental variable approach—we substantially strengthen the causal claim regarding digital literacy’s role. This provides robust, methodologically rigorous support for Norman and Skinner’s eHealth literacy framework (38) in the domain of physical activity, moving beyond correlational evidence.

### 4.2 Mechanisms underlying the suppression effect in rural contexts

The observed suppression effect regarding rural residents’ exercise duration—where digital access exerts a significant negative direct effect counterbalanced by a positive indirect effect via digital literacy—highlights the dual nature of technology as both a source of displacement and a potential health enabler. The negative direct path likely originates from time displacement and behavioral substitution. Here, unstructured digital engagement (e.g., entertainment-focused screen time) directly competes with and supplants time for physical activity, a phenomenon well-documented in sedentary behavior research (3, 39). This displacement may be particularly pronounced in rural settings due to a confluence of factors, including lower baseline health literacy, which can diminish the perceived priority of physical activity (15), and a digital environment where entertainment applications are often more immediately accessible and engaging than health-promoting content (19, 22). In contrast, the positive indirect pathway is activated through digital literacy. This competence, aligned with Social Cognitive Theory, equips individuals to transcend passive consumption by enabling them to seek, evaluate, and apply online health information effectively, thereby enhancing their health-related knowledge and outcome expectations. (17, 38). Furthermore, proficiency in using digital tools (e.g., fitness apps) increases self-efficacy for engaging in physical activity. Digitally literate individuals are also better positioned to leverage online communities for social support and normative influence, which facilitates the translation of intention into sustained behavior (4, 40). Consequently, the net positive total effect in rural areas materializes only when the empowering, literacy-mediated pathway outweighs the displacing direct effects of access. This finding underscores a critical policy implication: the mere provision of digital technology is insufficient without concurrently fostering the skills necessary to harness it for health.

### 4.3 Contextualizing the strength of the digital literacy effect

The finding that digital literacy exerts a stronger marginal effect on physical activity in rural compared to urban areas can be understood through the lens of relative resource scarcity and empowerment. In urban contexts, multiple avenues for promoting physical activity exist (e.g., gyms, parks, organized sports, abundant health information). Here, digital literacy is one facilitator among many. In contrast, rural areas often face constraints in physical infrastructure and formal health promotion resources. In such settings, digital literacy becomes a critical compensatory resource, unlocking a previously inaccessible world of health information, virtual guidance, and social connectivity related to fitness. The acquisition of this skill may therefore yield disproportionately large returns—a powerful empowerment effect—on health behavior in resource-constrained environments, explaining its larger estimated coefficient.

### 4.4 Policy implications and actionable recommendations

Our findings necessitate a strategic pivot in digital health policy—from a narrow focus on infrastructure to an integrated approach that concurrently builds digital capability and promotes health equity. To translate our empirical evidence on moderated mediation and suppression effects into concrete action, we propose a tripartite, context-specific action framework:

#### 4.4.1 Integrate access with contextualized literacy training to transform mechanisms

Policymakers must champion initiatives that bundle connectivity with compulsory, context-specific digital literacy training. This is particularly urgent in rural areas where our study identified significant suppression effects, indicating that access without literacy may displace physical activity. To transform access from a potential behavioral cost into a tool for empowerment, we recommend: (1) Co-designing “Digital Health Literacy” Curricula: Grounded in the eHealth Literacy Framework (38), curricula should focus on skills to locate, evaluate, and apply online health and fitness information. In rural contexts, training must explicitly aim to redirect digital engagement from passive entertainment towards active health management, thereby countering the negative direct path of the suppression effect. (2) Implementing Decentralized “Village Digital Health Ambassador” Programs: Inspired by community health worker models (41), training local volunteers can provide trusted, peer-level support. Ambassadors’ roles should extend beyond basic digital skills to include curating and demonstrating the use of specific health apps, accessing reliable local fitness resources online, and fostering online-offline activity groups. (3) Promoting Simplified and Localized Digital Health Tools: Support the development of health applications with interfaces compliant with universal design principles (42), featuring audio-visual guidance, local dialects, and low-data-usage options. This lowers the usage barrier, directly addressing the skill gap that underlies the rural suppression dynamic.

#### 4.4.2 Tailor resource allocation and programs to urban–rural contexts

A bifurcated strategy is essential to address the divergent mediation patterns (partial vs. suppression) and baseline disparities: 1) Urban Policies: Leverage higher baseline literacy and access by investing in advanced, personalized digital fitness ecosystems. This includes supporting platforms that integrate with wearable technologies, offer AI-driven coaching, and facilitate connections to local sports facilities and communities (4). The goal is to enhance the efficiency and personalization of the positive direct and indirect pathways already present. 2) Rural Interventions: Prioritize foundational skill development and infrastructure hybridization. This can be achieved by establishing offline-online hybrid community hubs. These hubs would provide: 1) Digital literacy labs with guided access to health content; 2) Spaces and equipment for physical activity (e.g., simple gyms, exercise yards); and 3) Organized sessions that explicitly link online learning with offline practice, such as following a workout video together. This model, aligned with WHO recommendations for resource-limited settings (43), directly tackles the resource scarcity that amplifies the marginal return on digital literacy in rural areas.

#### 4.4.3 Implement “smarter by design” regulation and nudges to shape digital environments

To directly counter the time displacement effects identified in our models—a key component of the rural suppression effect—we recommend shaping the digital environment itself: (1) Introduce Guidelines for Embedded Health Nudges: Regulatory bodies should encourage or mandate the integration of health-promoting nudges into commonly used applications and operating systems. Examples include: mandatory and actionable physical activity reminders after prolonged periods of passive screen time, or default settings that promote breaks. This applies insights from nudge theory (44) to mitigate the behavioral substitution risk associated with digital access. (2) Develop Age- and Literacy-Sensitive Design Standards: Establish regulatory standards requiring accessible design for public health and fitness applications. This includes mandating audio-visual tutorials, icon-based navigation, and compatibility with simple mobile phones to ensure inclusivity for older adults and those with lower literacy, leveraging evidence on senior technology adoption (45). (3) Foster “Data for Good” Initiatives: Encourage collaborations between public health bodies, tech companies, and researchers to utilize aggregated, anonymized data to identify “activity deserts” and tailor the promotion of localized, online-offline physical activity resources.

Ultimately, realizing the public health potential of digital technology requires transcending a narrow focus on mere access. It demands a synergistic strategy that cultivates contextualized digital literacy, tailors differentiated resources to specific populations, and responsibly shapes the digital environment through intelligent design—all to ensure equitable health outcomes.

### 4.5 Limitations and future research

This study has limitations. Its cross-sectional design limits definitive causal conclusions, though IV methods were used to mitigate this. The digital literacy index, while comprehensive, does not measure the specific purpose of device use (e.g., health vs. entertainment), an important dimension for future research. Self-reported physical activity measures may be subject to bias, and the use of hukou as a proxy for urban–rural context, while standard, may not capture residency mismatch. Future longitudinal studies should track the co-evolution of digital literacy and health behaviors, and qualitative work is needed to explore the lived experience of the digital displacement-empowerment dynamic in rural communities (41).

## 5 Conclusion

In conclusion, this study fundamentally reframes the relationship between digital technology and public health. We demonstrate that digital literacy, not mere access, is the critical lever for promoting physical activity in the digital age. The stark urban–rural heterogeneity in mechanisms—partial mediation versus suppression—reveals that the benefits of technology are not automatic but are filtered through and contingent upon existing socioeconomic structures and individual capabilities.

These findings necessitate a strategic policy pivot from a narrow focus on infrastructure expansion (the “digital access” agenda) to an integrated “digital capability” agenda. Public health strategies must concurrently provide access, build contextually relevant digital literacy, and shape healthier digital environments, particularly in rural areas where the risks of displacement are highest. Ensuring equitable digital literacy may prove to be as crucial for 21st-century public health as ensuring access itself.

## Data Availability

Publicly available datasets were analyzed in this study. This data can be found here: The datasets analyzed for this study can be found in the China Family Panel Studies (CFPS) repository, administered by the Institute of Social Science Survey (ISSS) at Peking University. The data are publicly available upon request at http://www.isss.pku.edu.cn/cfps/index.htm.

## Footnotes

1 Suppression Effect Interpretation: A suppression effect occurs when the direct effect of an independent variable (X) on a dependent variable (Y) and its indirect effect through a mediator (M) have opposite signs. In our rural context, digital access (X) has a negative direct effect on physical activity (Y), but a positive indirect effect through digital literacy (M). Think of digital literacy as a “filter” or “skill” that transforms the raw, potentially distracting nature of digital access into a useful tool for health. Without this filter, access may have a net negative impact; with it, the positive potential is unlocked, resulting in an overall positive relationship. The total effect is the sum of these countervailing direct and indirect paths.
