# Supplementary material for: Beyond ownership: the critical role of digital literacy in shaping the impact of digital access on physical activity
Source: Front Public Health. 2026 Jan 12;13:1718387. doi: 10.3389/fpubh.2025.1718387 (PMC12832747; doi:10.3389/fpubh.2025.1718387)
Supplement: Supplementary file 1 [file Supplementary_file_1.pdf]

Appendix A: Missing Data Rates and Final Sample Size Prior to Listwise Deletion

Table A1:Missing Data Rates and Final Sample Size Prior to Listwise Deletion

Variable	Obs.	Missing	Missing%
Exercise frequency	22,214	3,284	12.88%
Exercise duration	22,157	3,341	13.10%
Exercise participation	22,326	3,172	12.44%
Digital Access	22,614	2,884	11.31%
Digital literacy	22,613	2,885	11.31%
Hukou	22,675	2,823	11.07%
Gender	25498	0	0.00%
Age	25498	0	0.00%
partnered	22,614	2,884	11.31%
Years of education	22,848	2,650	10.39%
Ln household income	25498	0	0.00%
Subjective social status	22,156	3,342	13.11%
Self-rated health	25,096	402	1.58%
Chronic disease	22,607	2,891	11.34%
Region	25498	0	0.00%
Final Analytical Sample	18251	7247	28.42%

Note. This table reports the prevalence of missing data for all variables in the initial pooled dataset (N = 25,498). The missing rate for individual variables ranged from 0% to 13.11%. The application of listwise deletion, which excluded observations with any missing values on the core variables (digital access, digital literacy, physical activity, and hukou) or controls, resulted in a final analytical sample of 18,251 complete cases (71.58% of the initial sample). We acknowledge the non-trivial cumulative missingness (28.42%) and have mitigated potential selection bias through comprehensive covariate adjustment for factors associated with missingness (e.g., age, education, income) and demonstrated the robustness of our core findings across multiple model specifications and sub-samples, as detailed in the main text.
