# Supplementary material for: Beyond ownership: the critical role of digital literacy in shaping the impact of digital access on physical activity
Source: Front Public Health. 2026 Jan 12;13:1718387. doi: 10.3389/fpubh.2025.1718387 (PMC12832747; doi:10.3389/fpubh.2025.1718387)
Supplement: Supplementary file 2 [file Supplementary_file_2.pdf]

Appendix B: Digital-literacy Index Construction

1. Measurement Framework of Digital Literacy Indicators

The digital literacy index was constructed using 12 items from the 2022 China Family Panel Studies (CFPS), grouped into five dimensions. Each item was normalized and weighted using Shannon's entropy algorithm. The table below details the measurement items, scoring rules, and entropy weights:

Table B1:Digital Literacy Measurement Framework

Dimension	Measurement Item	Scoring Rules	Weight
Digital Usage	Combined duration of mobile and computer internet usage (hours/week)	Sum of qu201a (mobile) and qu202a (computer); missing values (-8) set to 0; winsorized at 98th percentile	0.097
Digital Learning	1. Importance of internet for learning	qu954: 1 (very unimportant) to 5 (very important); missing values (-8) set to 0	0.042
	2. Online learning behavior	qu94 (yes=1, no=0) and qu941 (daily=2, occasional=1); Composite: Daily=2, occasional=1, no=0	0.200
Digital Social	1. Importance of internet for family/friend contact	qu953: 1 (very unimportant) to 5 (very important); missing values (-8) set to 0	0.038
	2. Use of WeChat	qu11: yes=1, no=0; missing values (-8) set to 0	0.039
	3. Frequency of sharing on WeChat Moments	qu111: 1 (never) to 7 (almost daily); reverse-coded (8-q111); missing values (-8) set to 7	0.113
Digital Work	Importance of internet for work	qu951: 1 (very unimportant) to 5 (very important); missing values (-8) set to 0	0.045
Digital Life	1. Importance of internet for daily life	qu955: 1 (very unimportant) to 5 (very important); missing values (-8) set to 0	0.045
	2. Importance of internet for leisure/entertainment	qu952: 1 (very unimportant) to 5 (very important); missing values (-8) set to 0	0.043
	3. Online shopping	qu92 (yes=1, no=0) and qu921 (daily=2, occasional=1); Composite: Daily=2, occasional=1, no=0	0.119
	4. Watching short videos	qu93 (yes=1, no=0) and qu931 (daily=2, occasional=1); Composite: Daily=2, occasional=1, no=0	0.015
	5. Online gaming	qu91 (yes=1, no=0) and qu911 (daily=2, occasional=1); Composite: Daily=2, occasional=1, no=0	0.204

2. Entropy Weight Method: Computational Steps

The entropy weight method objectively assigns weights based on the variability of each indicator. Higher variability implies greater informational value and thus a higher weight. Steps are as follows:

Step 1: Normalization

For each of the 12 items x_i, min-max normalization was applied to rescale values to [0,1]:

$$sx_i = \frac{x_i - \min(x_i)}{\max(x_i) - \min(x_i)}$$

Values of 0 were replaced with 0.0001 to avoid undefined logarithms.

Step 2: Calculate Probability p_i

For each observation j of item i:

$$p_{ij} = \frac{sx_{ij}}{\sum_{j=1}^{n} sx_{ij}} \quad (n = sample\ size)$$

Step 3: Compute Entropy e_i

$$e_i = -\frac{1}{\ln(n)} \sum_{j=i}^{n} p_{ij} \ln(p_{ij})$$

Step 4: Calculate Information Utility d_i

$$d_i = 1 - e_i$$

Step 5: Determine Weight w_i

$$w_i = \frac{d_i}{\sum_{i=1}^{12} d_i}$$

Step 6: Composite Score

The digital literacy index for each individual is the weighted sum:

$$Digital\ Literacy\ Score = \sum_{i=1}^{12} w_i \cdot sx_i$$

3. Stata Implementation Code

Step 1: Normalization and winsorization

```
global items x1 x2 x3 x4 x5 x6 x7 x8 x9 x10 x11 x12
foreach var in $items {
    sum `var', detail
    gen s`var' = (`var' - r(min)) / (r(max) - r(min))
    replace s`var' = 0.0001 if s`var' == 0
}
```

Step 2: Probability matrix

```
forval i = 1/12 {
    egen sum_sx`i' = sum(sx`i')
    gen p`i' = sx`i' / sum_sx`i'
}
```

Step 3: Entropy calculation

```
forval i = 1/12 {
    gen temp`i' = p`i' * ln(p`i')
    egen sum_temp`i' = sum(temp`i')
    gen e`i' = -sum_temp`i' / ln(18336)    // n=18,336
    gen d`i' = 1 - e`i'
}
```

Step 4: Entropy weights

```
egen total_d = rowtotal(d1-d12)
forval i = 1/12 {
    gen w`i' = d`i' / total_d
}
```

Step 5: Composite score

```
forval i = 1/12 {
    gen score_part`i' = w`i' * sx`i'
}
egen digital_literacy = rowtotal(score_part1-score_part12)
```

4. Validation

Weights: Sum to 1.000 (validation: =0.097+0.042+⋯ +0.204=1).

Index Range: 0 (lowest) to 1 (highest digital literacy).

Sensitivity: Weights are data-driven, reflecting each indicator’s discriminative power.

5. Interpretation of Entropy Weights

The entropy method objectively assigns weights based on an indicator's informational value

(variability). The resulting weights, while data-driven, warrant theoretical interpretation. The high weights assigned to Online Learning (0.200) and Online Gaming (0.204) suggest that these indicators are particularly effective in discriminating between individuals' digital competency levels in our sample. This is conceptually coherent: both activities represent active, skilled engagement with digital technology—requiring information seeking, processing, and often strategic interaction. They likely tap into higher-order dimensions of digital literacy beyond mere access or basic usage. In contrast, Internet Usage Duration (0.097), while fundamental, is a more generic measure of exposure that may exhibit less variation in a population with widespread basic connectivity. Thus, the entropy-weighted composite index aligns with a nuanced understanding of digital literacy that emphasizes the depth, diversity, and complexity of technology use over simple duration.
