# Supplementary material for: Beyond ownership: the critical role of digital literacy in shaping the impact of digital access on physical activity
Source: Front Public Health. 2026 Jan 12;13:1718387. doi: 10.3389/fpubh.2025.1718387 (PMC12832747; doi:10.3389/fpubh.2025.1718387)
Supplement: Supplementary file 3 [file Supplementary_file_3.pdf]

Appendix C: Sensitivity Analysis for Causal Mediation on a 50% Random Subsample

Table C1 Sensitivity Analysis: Causal Mediation of Digital Access on Physical Activity via Digital Literacy (50% Random Subsample, N = 9,125)

Model<br>Variable	(1)<br>Digital Literacy	(2)<br>Exercise Frequency	(3)<br>Exercise Duration
**Stepwise Regression Coefficients**			
Digital Access	0.190*** (0.003)	0.055 (0.079)	-1.306** (1.165)
Digital Literacy	–	1.551*** (0.207)	27.090*** (3.048)
**Causal Mediation Analysis***(Imai et al., 2010 Framework)*			
ACME (Indirect Effect)	–	**0.297**[0.228, 0.374]	**5.144**[4.335, 5.935]
ADE (Direct Effect)	–	**0.050**[-0.102, 0.196]	**-1.885**[-3.418, -0.330]
Total Effect	–	**0.346**[0.214, 0.478]	**3.259**[1.901, 4.643]
Proportion Mediated	–	**85.9%**[0.621, 1.389]	**157.9%**[1.108, 2.706]
**Sensitivity results (95% Confidence interval)**			
Rho at which ACME = 0		0.0781	0.0927
R^2_M*R^2_Y* at which ACME = 0		0.0061	0.0086
R^2_M~R^2_Y~ at which ACME = 0		0.0019	0.0026
**Model Information**			
Controls	Yes	Yes	Yes
Regional Effects	Yes	Yes	Yes
_cons	0.145*** (0.014)	-1.701*** (0.283)	-18.468*** (2.808)
N	9,125	9,125	9,125
r^2	0.6598	0.1003	0.1137

Note. This table presents the causal mediation analysis and sensitivity check conducted on a randomly selected 50% subsample (N = 9,125). The analysis was performed to assess the robustness of the full-sample findings (Table 4) and to enable the sensitivity analysis, which was computationally prohibitive for the full sample. The key estimates for the **ACME**, **ADE**, and **Total Effect** in this subsample are remarkably consistent with the full-sample results, confirming the robustness of the identified mediation and suppression mechanisms. The sensitivity parameters (ρ) indicate that an unobserved confounder would need to be correlated with the residuals of both the mediator and outcome at a level of ρ > 0.078 (for frequency) and ρ > 0.093 (for duration) to explain away the estimated mediation effects. This provides strong evidence for the robustness of our conclusions to potential omitted variable bias.
