# Supplementary material for: Beyond ownership: the critical role of digital literacy in shaping the impact of digital access on physical activity
Source: Front Public Health. 2026 Jan 12;13:1718387. doi: 10.3389/fpubh.2025.1718387 (PMC12832747; doi:10.3389/fpubh.2025.1718387)
Supplement: Supplementary file 4 [file Supplementary_file_4.pdf]

Appendix D: Robustness Checks Using Ordered Logit Models for Exercise Frequency

Table D1. Robustness Check: Ordered Logit Regression for Exercise Frequency (Full Sample)

Ordered logistic regression — Number of obs = 18,251
Wald chi2(13) = 2135.29
Prob > chi2 = 0.0000
Log pseudolikelihood = -24831.045 — Pseudo R2 = 0.0438

Exercise frequency	Coefficient	Robust std. err.	z	P>z	[95% conf. interval]	
Digital Access	0.1239	0.0560	2.210	0.027	0.0143	0.2336
Digital literacy	1.3712	0.1205	11.380	0.000	1.1351	1.6073
Hukou	0.5280	0.0329	16.030	0.000	0.4634	0.5925
Gender	0.0686	0.0291	2.360	0.018	0.0116	0.1257
Age	0.0241	0.0015	15.650	0.000	0.0211	0.0271
partnered	-0.5974	0.0352	-16.950	0.000	-0.6665	-0.5283
Years of education	0.0825	0.0046	17.880	0.000	0.0734	0.0915
Ln household income	0.0606	0.0165	3.680	0.000	0.0284	0.0929
Subjective social status	0.0813	0.0158	5.160	0.000	0.0504	0.1123
Self-rated health	0.1168	0.0150	7.800	0.000	0.0875	0.1462
Chronic disease	0.3329	0.0480	6.940	0.000	0.2389	0.4269
East	-0.1582	0.0345	-4.590	0.000	-0.2257	-0.0907
West	-0.0162	0.0391	-0.420	0.678	-0.0928	0.0603
/cut1	3.6622	0.1846			3.3004	4.0240
/cut2	3.7621	0.1848			3.3999	4.1242
/cut3	4.0411	0.1852			3.6782	4.4041
/cut4	4.6665	0.1861			4.3017	5.0313
/cut5	5.0505	0.1869			4.6843	5.4168
/cut6	5.1536	0.1870			4.7870	5.5202
/cut7	7.1605	0.1943			6.7797	7.5413

Note. This table presents the results of an ordered logit regression on exercise frequency for the full sample (N = 18,251), conducted to verify the robustness of our primary OLS estimates to the ordered categorical nature of the outcome variable. The key findings are fully consistent with the main analysis: **digital literacy exhibits a strong, positive, and highly statistically significant association with exercise frequency** (Coeff. = 1.371, $p < 0.001$), reinforcing its role as the primary driver. The positive and significant coefficient for digital access (Coeff. = 0.124, $p < 0.05$) aligns with the positive total effect identified in the causal mediation analysis.

Table D2. Robustness Check: Ordered Logit Regression for Exercise Frequency (Urban Sub-Sample)

Ordered logistic regression Number of obs = 5,428

Wald chi2(12) = 419.83

Prob > chi2 = 0.0000

Log pseudolikelihood = -8965.3457 Pseudo R2 = 0.0264

Exercise frequency	Coefficient	Robust std. err.	z	P>z	[95% conf. interval]	
Digital Access	0.2753203	0.1131437	2.43	0.015	0.0535627	0.4970779
Digital literacy	0.545489	0.1932851	2.82	0.005	0.1666573	0.9243208
Gender	0.2165243	0.0498393	4.34	0.000	0.1188411	0.3142075
Age	0.0344031	0.0025178	13.66	0.000	0.0294683	0.0393379
partnered	-0.5045061	0.0634873	-7.95	0.000	-0.6289389	-0.3800732
Years of education	0.073026	0.0084824	8.61	0.000	0.0564009	0.0896512
Ln household income	0.129183	0.0283676	4.55	0.000	0.0735835	0.1847826
Subjective social status	0.0919627	0.0304379	3.02	0.003	0.0323055	0.1516198
Self-rated health	0.1738022	0.0281026	6.18	0.000	0.1187221	0.2288823
Chronic disease	0.3021152	0.0780679	3.87	0.000	0.1491048	0.4551255
East	-0.1895485	0.0573264	-3.31	0.001	-0.3019062	-0.0771908
West	-0.0026529	0.0718886	-0.04	0.971	-0.143552	0.1382461
/cut1	4.253197	0.3214627			3.623141	4.883252
/cut2	4.365467	0.3218454			3.734662	4.996273
/cut3	4.67497	0.322919			4.04206	5.307879
/cut4	5.366528	0.3251537			4.729239	6.003818
/cut5	5.808839	0.3268367			5.168251	6.449427
/cut6	5.932164	0.3274808			5.290313	6.574014
/cut7	7.957381	0.3381811			7.294558	8.620204

Note. Ordered logit regression results for the urban sub-sample (N = 5,428). The results corroborate the **partial mediation** pattern identified in the main text. Both **digital access** (Coeff. = 0.275, $p < 0.05$) and **digital literacy** (Coeff. = 0.545, $p < 0.01$) show significant positive coefficients, indicating that each factor independently contributes to higher exercise frequency among urban residents, consistent with the findings from our primary causal mediation analysis.

Table D3. Robustness Check: Ordered Logit Regression for Exercise Frequency (Rural Sub-Sample

Ordered logistic regression Number of obs = 12,823

Wald chi2(12) = 1171.48

Prob > chi2 = 0.0000

Log pseudolikelihood = -15732.433 Pseudo R2 = 0.0339

Exercise frequency	Coefficient	Robust std. err.	z	P>z	[95% conf. interval]	
Digital Access	-0.064354	0.0655253	-0.98	0.326	-0.1927813	0.0640732
Digital literacy	1.998875	0.1533084	13.04	0.000	1.698396	2.299354
Gender	0.0002316	0.0360954	0.01	0.995	-0.0705141	0.0709772
Age	0.0168868	0.0020199	8.36	0.000	0.012928	0.0208457
partnered	-0.6198745	0.042455	-14.6	0.000	-0.7030848	-0.5366643
Years of education	0.077306	0.0056385	13.71	0.000	0.0662546	0.0883573
Ln household income	0.0195179	0.0198831	0.98	0.326	-0.0194523	0.0584881
Subjective social status	0.0894239	0.0186075	4.81	0.000	0.0529538	0.1258939
Self-rated health	0.0840249	0.0180069	4.67	0.000	0.048732	0.1193178
Chronic disease	0.3450149	0.0619831	5.57	0.000	0.2235303	0.4664994
East	-0.1400436	0.0435614	-3.21	0.001	-0.2254223	-0.0546649
West	-0.0374327	0.0469905	-0.8	0.426	-0.1295324	0.0546669
/cut1	2.858899	0.2315825			2.405005	3.312792
/cut2	2.954272	0.2317052			2.500139	3.408406
/cut3	3.221844	0.231931			2.767268	3.676421
/cut4	3.818115	0.2324783			3.362466	4.273764
/cut5	4.172087	0.2330347			3.715347	4.628827
/cut6	4.263969	0.2330073			3.807283	4.720655
/cut7	6.280062	0.2430563			5.80368	6.756444

Note. Ordered logit regression results for the rural sub-sample (N = 12,823). The results provide strong evidence for the **suppression effect**. While **digital literacy remains powerfully positive and significant** (Coeff. = 1.999, $p < 0.001$), the **direct coefficient for digital access is negative and statistically insignificant** (Coeff. = -0.064, p = 0.326). This pattern, where the positive effect of access is fully channeled through literacy with no independent direct benefit, is a hallmark of suppression and perfectly aligns with the mechanisms uncovered by our primary models.
